# COVID-19 Pandemic and Sleep Health in Polish Female Students

**DOI:** 10.3390/jcm14155342

**Published:** 2025-07-29

**Authors:** Mateusz Babicki, Tomasz Witaszek, Agnieszka Mastalerz-Migas

**Affiliations:** 1 Department of Family Medicine, Faculty of Medicine, Wroclaw Medical University, 50-367 Wroclaw, Poland; agnieszka.mastalerz-migas@umw.edu.pl; 2 Tomasz Witaszek—Indywidualna Praktyka Lekarska, ul. Józefińska 33/8, 30-529 Krakow, Poland; witaszek.tomasz@gmail.com

**Keywords:** sleep disorders, insomnia, quality of life, students, young adults, COVID-19

## Abstract

**Background**: Insomnia and excessive sleepiness are significant health problems with a complex etiology, increasingly affecting young people, especially students. This study aimed to assess the prevalence of sleep disturbances and patterns of psychoactive drug use among female Polish students. We also explored the potential impact of the COVID-19 pandemic on sleep behaviors. We hypothesized that sleep disorders are common in this group, that medical students are more likely to experience insomnia and excessive sleepiness, and that the pandemic has exacerbated both sleep disturbances and substance use. **Methods**: This cross-sectional study utilized a custom survey designed using standardized questionnaires—the Athens Insomnia Scale and Epworth Sleepiness Scale—that was distributed online using the Computer-Assisted Web Interviewing method. A total of 11,988 responses were collected from 31 January 2016 to 1 January 2021. Inclusion criteria were being female, having a college student status, and giving informed consent. **Results**: Among the 11,988 participants, alcohol use declined after the pandemic began (*p* = 0.001), while sedative use increased (*p* < 0.001). Insomnia (AIS) was associated with study year, university profile, and field of study (*p* < 0.001), with the highest rates in first-year and non-medical students. It was more common among users of sedatives, psychostimulants, and multiple substances. No significant change in insomnia was found before and after the pandemic. Excessive sleepiness (ESS) peaked in first-year and medical students. It decreased during the pandemic (*p* < 0.001) and was linked to the use of alcohol, psychostimulants, cannabinoids, and multiple substances. **Conclusions**: These findings highlight that female students are particularly vulnerable to sleep disorders. The influence of the COVID-19 pandemic on sleep disturbances remains inconclusive. Given the varied results in the existing literature, further research is needed.

## 1. Introduction

Insomnia and excessive daytime sleepiness (EDS) are two of the most common sleep-related issues globally, affecting an increasing number of people [1]. Insomnia is characterized by difficulty initiating or maintaining sleep, or non-restorative sleep, despite adequate opportunity to sleep [2]. EDS refers to an increased propensity to fall asleep during daytime activities and is often a symptom of underlying sleep problems [3]. Both conditions impair physical, mental, and social functioning, reducing quality of life [4]. In recent years, they have become increasingly prevalent among young people, particularly students, with some studies showing that over 70% sleep less than 7 h per night during the school week [5].

A comprehensive 2023 meta-analysis of 95 studies involving 54,894 medical students reported that 55.6% experienced poor sleep quality and 33.32% reported excessive daytime sleepiness [6], supporting earlier findings from a 2021 systematic review showing that students have poorer sleep health than the general population, with medical students being the most affected subgroup [7]. Similarly, Jiang et al. (2015) reported an insomnia prevalence of 18.5% (95% CI: 11.2–28.8%) among university students—considerably higher than the 7.4% reported in the general population [8]. Additionally, a meta-analysis of 13 studies examining gender differences found that females had a significantly higher prevalence of insomnia compared to males [9].

The period of studies involves significant life changes, which can often impact circadian rhythms. Lack of parental control and becoming more independent, moving to a new place, changing peer groups, and trying to ‘fit in’ or taking on new responsibilities can all affect sleep quality [10]. Additionally, during this period, young adults may experience Delayed Sleep Phase Disorder (DSPD), characterized by a delay in the main sleep episode, where most of the activity takes place in the evening and the night, and morning becomes a period of sleep and rest [11]. Students are also more prone to chronic sleep deprivation caused by waking up early every day [12]. Inadequate sleep in students has been associated with poorer academic performance, increased risk of mental health disorders, and unhealthy coping strategies, such as substance use [13,14,15,16].

Research shows that, in the general population, women are more often affected by sleep disorders than men, regardless of age or background [17,18], largely due to hormonal factors. The menstrual cycle alters circadian rhythms and sleep patterns, with many women reporting poorer sleep and more disturbances during the premenstrual phase. Those with severe premenstrual syndrome often experience disturbing dreams, fatigue, and decreased alertness [19].

Despite reporting more sleep problems, young women do not show poorer sleep quality on objective tests, suggesting mood disorders may contribute [20]. Among students, sleep issues often co-occur with mental health problems like depression, stress, and anxiety [21]. Depression and sleep disturbances likely interact bidirectionally, though the relationship is complex—acute sleep deprivation, for example, can sometimes improve depressive symptoms [22].

The negative impact of the COVID-19 pandemic on sleep quality and overall quality of life has been well documented [23,24]. An increase in sleep disturbances has been observed both in the general population and among students. Studies showed that the COVID-19 pandemic increased daytime sleepiness, worsening the quality of sleep without increasing the phenomenon of general insomnia [25]. Adverse psychological outcomes of the COVID-19 pandemic, such as anxiety, depression, sleep disorders, and post-traumatic stress disorder, more often affect younger adults, including university students [26].

Establishing the epidemiology of sleep disturbances is challenging due to group differences and a lack of large-scale studies. Existing research often shows inconsistent results. The wide range of prevalence rates, variations in sample and methodology, and risk of publication bias suggest these estimates should be interpreted with caution. For example, one meta-analysis reported that poor sleep quality in adults ranged from 6% to 94%, depending on the study [27]. This highlights the need for more comprehensive research.

Our study aimed to assess insomnia and daytime sleepiness among female Polish students, considering their field of study, year in university, and patterns of psychoactive drug use. Additionally, it examines the potential impact of the COVID-19 pandemic on sleep patterns within this population.

The authors formulated the following research hypotheses: (1) Sleep disorders are common among Polish female students. (2) Medical students suffer from insomnia and daytime sleepiness more frequently than their peers. (3) The COVID-19 pandemic would exacerbate sleep disorders and psychoactive drug use among students.

## 2. Materials and Methods

### 2.1. The Methodology

This cross-sectional study utilized a custom survey designed using standardized questionnaires. It was distributed online from 31 January 2016 to 1 January 2021 through student-associated social network groups on Facebook. In most cases, the students’ status was confirmed via their membership. The survey was administered to female students living and studying in Poland. Participation in the survey was anonymous and voluntary. We established 11 March 2020 as the starting date of the COVID-19 pandemic (based on the WHO announcement) [28]; therefore, responses were grouped as either pre-pandemic (before 11 March 2020) or post-pandemic onset (on or after 11 March 2020).

The main study variables were insomnia symptoms, measured using the Athens Insomnia Scale (AIS), and daytime sleepiness, measured using the Epworth Sleepiness Scale (ESS). The secondary study variables included sociodemographic characteristics (age, place of residence, field and year of study), frequency of psychoactive substance use (alcohol, cannabinoids, psychostimulants, and hypnotics), and the timing of survey completion in relation to the COVID-19 pandemic.

Inclusion criteria were being female, having a college student status, and providing informed consent. Exclusion criteria included being under 18 years of age and providing incomplete or duplicate responses.

After being introduced to the research objectives and methodology, the respondents were asked to provide informed consent to participate in the study. At any point, participants could terminate their participation without giving any reason. Consenting participants were then requested to continue with the study procedure.

The survey questionnaire consisted of two parts. The first part assessed the socio-demographic status of the respondents (including age, sex, place of residence, field of study, and year of study) and the frequency of psychoactive substance use (alcohol, cannabinoids, psychostimulants, and hypnotics). The second part involved standardized psychometric tools: the Athens Insomnia Scale (AIS) and the Epworth Sleepiness Scale (ESS).

The study was conducted according to the guidelines of the Declaration of Helsinki and was approved by the Bioethics Committee of the Wroclaw Medical University, Poland (approval number KB-234/2021, issued on 18 March 2021). In Poland, ethical approval is not required for anonymous, observational survey studies without interventions.

### 2.2. Athens Insomnia Scale (AIS)

The AIS scale is an 8-item validated questionnaire designed to assess insomnia symptoms based on the ICD-10 criteria [29]. It employs a four-point Likert scale (0—no difficulty, 3—severe difficulty) to evaluate issues with falling asleep, waking up during the night, waking up early in the morning, total sleep time, sleep quality, well-being the next day, mental and physical alertness the next day, and daytime sleepiness occurring at least three times in the last 14 days. The maximum score achievable is 24. The Polish validated adaptation of the AIS was used, with a diagnostic threshold set at 8 points for diagnosing insomnia [30]. This measurement demonstrates high sensitivity (93%) and specificity (85%). Additionally, Cronbach’s alpha indicated high reliability, with a value of 0.862.

### 2.3. Epworth Sleepiness Scale (ESS)

The ESS scale is the most commonly used validated tool to measure excessive sleepiness and to diagnose sleepiness [31]. It consists of 8 questions assessing the likelihood of falling asleep in various scenarios, such as watching TV, sitting in a public place, lying down, and relaxing in the afternoon. The maximum score is 24. In this study, the following scoring criteria were used: 0–10 points indicate no daytime sleepiness, 11–14 points indicate sleepiness, and 15 points and above indicate pathological sleepiness, suggesting consultation with a doctor. The Cronbach’s alpha coefficient was calculated to be 0.798.

### 2.4. Statistical Analysis

The analysis pertained to qualitative and quantitative variables. Basic descriptive statistics were used. The normality of the variables was assessed using the Shapiro–Wilk test. The Chi-square test was used to assess differences between qualitative variables. The Mann–Whitney U test was used for quantitative variables. The Statistica program (version 13.3) was used to compute *t*-test and chi-square statistics.

## 3. Results

### 3.1. Characteristics of the Study Group

A total of 11,988 Polish female students participated in the study. The average age was 22 years old. The vast majority (76.1%, n = 9127) of participants were students of non-medical universities. A total of 51.1% (n = 6129) of students completed the survey before the outbreak of the COVID-19 pandemic.

A total of 89.7% (n = 10,759) of students used some form of stimulants, with alcohol being the most popular. Interestingly, alcohol was used significantly less after the COVID-19 pandemic announcement (*p* = 0.001), while sedatives were used more frequently (*p* < 0.001). However, the general trend was that the use of substances decreased after the pandemic started (*p* < 0.001). A detailed comparison of the entire study group and a comparison with regard to the COVID-19 pandemic is presented in Table 1.

### 3.2. Assessments of Insomnia and Excessive Sleepiness

Using the AIS, insomnia rates were found to be significantly associated with the study year, university profile, and the field of study (*p* < 0.001 for all). The prevalence of insomnia decreased over the initial years of university, followed by a marked increase in the final year. Non-medical students reported higher insomnia rates overall, with the highest prevalence observed among students in the biological sciences. Both the use of sedatives and their frequency were also related to insomnia (*p* < 0.001). People who used more substances suffered from insomnia more frequently (*p* < 0.001). There was no significant difference in insomnia rates before and after the COVID-19 pandemic announcement. A more detailed assessment of the effects of university profile, the pandemic, and psychoactive substance use on insomnia using the Athens Insomnia Scale (AIS) can be found in Table 2.

Using the ESS, excessive daytime sleepiness was significantly associated with the study year (*p* = 0.024), university profile, and the field of study (*p* < 0.001 for both). Similar to insomnia, its prevalence decreased over the course of the study before peaking in the final year. Medical students reported higher levels of excessive sleepiness overall, with the highest rates observed among pharmacy students. Interestingly, the COVID-19 pandemic had a significant impact on excessive sleepiness rates, which decreased during the pandemic period (*p* < 0.001). A statistically significant association was found between the use of any psychoactive substance and excessive sleepiness; however, no relationship was observed with the frequency of use. Excessive sleepiness was more frequently reported among individuals using alcohol, psychostimulants, and cannabinoids. A more detailed assessment of the effects of university profile, the pandemic, and psychoactive substance use on excessive sleepiness using the Epworth Sleepiness Scale (ESS) can be found in Table 3.

## 4. Discussion

This study aimed to assess the prevalence of insomnia and excessive daytime sleepiness among Polish female college students, with particular attention to potential differences before and after the announcement of the COVID-19 pandemic. In the academic year 2024/2025, 58.2% of the 1.28 million university students in Poland were women, with the most common fields among female graduates being business, administration and law, health and social care, and social sciences [32]. While our sample does not fully reflect the national distribution across disciplines, the overrepresentation of female students is partly attributable to their greater willingness to participate in anonymous online surveys, a trend observed in similar research.

Sleep disorders are an increasingly significant global health concern, with the number of affected individuals rising each year. Our findings highlight that students, in particular, represent a group at heightened risk for developing such disorders. Insomnia is commonly referred to as the most prevalent sleep problem among students [33]. However, in the existing literature, its rates vary from as high as 61.6% among Ethiopian students [34] to 7.7% among German students [35]. Thus, its prevalence varies depending on the population surveyed, the time of data collection, and the use of different validated tools. Individuals in lower socioeconomic status groups are also thought to be at risk for sleep disturbances and associated health consequences [36]. Both insomnia and excessive sleepiness were more prevalent during the early years of university. This is consistent with previous research; for example, Goweda et al. found that the rates of sleep disorders ranged from 88.6% in the second year to 65% in the sixth year [37]. Studies indicate that stress levels are highest in the first year of university and gradually decrease as students adapt to the workload and their new routines [38]. When examining the field of study, medical students had lower rates of insomnia but higher rates of excessive sleepiness compared to their peers. This finding somewhat contradicts most research, which typically shows that medical students report the highest rates of disrupted sleeping patterns [8,39]. Although sleep quality is a concern across all fields of study, medical students may be particularly vulnerable due to the unique demands of medical education. They face high academic demands, long study and clinical hours, and other factors that can cause sleep disturbances and disorders [6]. In our study, the COVID-19 pandemic announcement did not impact insomnia rates, but it did lower excessive sleepiness. Given the timeline of our data collection, it is important to note that the data were gathered during the lockdown, with most university activities conducted through remote study platforms and outside activity severely limited. Meta-analyses indicate that the pandemic indeed led to changes in sleep patterns [40]. Several studies show an increase in sleep duration, with participants going to bed later, getting out of bed later, and taking more naps during the day [41,42]. However, nearly one in five participants reported a decrease in their sleep duration, and the results varied depending on factors such as their place of residence [40]. This highlights a significant discrepancy in the available data, making it difficult to draw definitive conclusions. Fewer external demands and more flexible daily schedules may have helped students sleep in a way that better matched their personal chronotypes, leading to less daytime sleepiness. However, ongoing stress and a sense of uncertainty may have kept insomnia levels high despite these changes.

Given the study’s focus on female students, it is important to consider gender-specific factors that may influence the prevalence of sleep disorders. Women have been reported to experience sleep disorders more frequently than men [9]. Nowicki et al. found that, among the general population of Poland, the prevalence of self-reported insomnia was 58.9% in women and 41.4% in men [43]. Psychological and hormonal factors can influence this phenomenon. Women are at a higher risk of developing psychiatric disorders such as depression or generalized anxiety disorder, and these conditions have been linked to sleep disturbances [44]. This higher level of heightened emotional responsiveness can be explained by differences in sex steroid hormone levels between sexes after puberty [45]. In women, hormone levels fluctuate with the menstrual cycle, with documented impacts on self-assessed quality and duration of sleep [46,47]. Some studies have reported that high estradiol and progesterone levels are associated with prolonged arousal and wakefulness [48].

Substance use and sleep patterns are bidirectionally linked, influencing each other in significant ways. Participants who used psychostimulants and sedatives were more frequently diagnosed with insomnia. For psychostimulant users, sleep disturbances are often a direct consequence of substance use, as studies have shown that psychostimulant consumption negatively impacts sleep quality [15]. In a study of 498 adolescent participants aged 18 to 25, Pittsburgh Sleep Quality Index (PSQI) global scores were positively and significantly associated with the number of cigarettes smoked per day and the number of days of non-medical prescription stimulant (NPS) use [49]. It is important to note that the relationship between stimulants and sleep is complex, with studies offering conflicting evidence. Additionally, sedative users might have initially turned to these substances to address pre-existing insomnia. While research indicates that non-medical use of prescription sedatives can decrease sleep quality in students [50], it also suggests that sedatives can improve sleep quality in other populations when comparing effects before and after use [51]. In our study, the impact of substances on excessive daytime sleepiness is evident, with every substance showing a significant relationship with its prevalence. This bidirectional relationship suggests that sleep and alertness alterations, while not the primary reinforcing mechanism, may contribute to the initiation and maintenance of drug and alcohol abuse and increase the risk of relapse [52].

The COVID-19 pandemic influenced substance use, which may be connected to changes in the rates of sleep disturbances. The results of this study suggest that students were most likely to consume alcohol both before and during the pandemic. However, they consumed alcohol much less frequently during the initial phase of the pandemic. This aligns with studies suggesting that more Europeans limited their alcohol consumption when the pandemic started [53,54]. As most students consume alcohol in social settings [55], it could explain why isolation affected its intake. It is worth noting that, despite the general trend, some studies found that 14–16% of adults reported an increase in their intake [56]. Another study found that, in the early stages of the pandemic, one in four students surveyed developed riskier alcohol-related behaviors [57]. Sedative use increased after the start of the pandemic, which aligns with findings from most studies [58]. This rise may be attributed to heightened anxiety levels during lockdown [59,60], which can significantly disrupt sleep patterns, leading to issues like insomnia and a greater reliance on medication. Interestingly, some studies also report an increase in health-promoting behaviors, such as higher levels of physical activity, increased consumption of fruits and vegetables, and reduced tobacco use [61]. It is possible that the fear of the virus and death during the COVID-19 pandemic motivated these healthier habits. However, substance use patterns during the pandemic appear to vary based on factors like location, time period, and individual predispositions. Therefore, ongoing monitoring is essential.

We acknowledge several limitations of this study, including the data collection methodology, which involved using an anonymous online questionnaire. While this approach allows for reaching a large audience and increases survey participation rates, it also presents challenges. The data are cross-sectional, capturing a single point in time. Therefore, causal relationships between variables cannot be established. Prior research indicates that anonymous online surveys can significantly reduce respondents’ anxiety and boost the likelihood of participation [62]. However, such surveys do not allow the authors to verify the accuracy of the provided data, determine the response rate, and limit the ability to fully verify participants’ eligibility and prevent duplicate or inauthentic responses. The data collection spanned five years, covering different social, academic, and epidemiological contexts, and the analysis did not specifically address this temporal variation. The psychometric tools used rely on respondents’ subjective assessments and require verification and objectification by a trained medical professional. The survey did not include questions regarding sleep disorders confirmed by a medical specialist, nor did it assess whether participants were undergoing treatment for such conditions. It also did not include questions about prior COVID-19 infection, which is known to potentially affect brain activity and sleep patterns [63]. The study did not include objective diagnostic methods such as polysomnography, which remains the gold standard for the assessment of sleep disorders [64]. The interpretation of this study’s findings is further limited by the lack of data on chronic conditions, chronic medication use, and potential mental health issues within the surveyed population. However, it should be noted that the psychometric tools employed are recognized as valid and effective research instruments. Although the Pittsburgh Sleep Quality Index (PSQI) is a widely used tool for assessing sleep quality, we chose the Athens Insomnia Scale (AIS) and the Epworth Sleepiness Scale (ESS) for this study because they specifically target insomnia symptoms and daytime sleepiness. Both instruments are concise, validated, and well-suited for large-scale online surveys. Due to the exploratory nature and methodological constraints of our study, we focused on univariate and bivariate associations, which do not account for potential confounding. Future research should incorporate multivariate models to better assess independent associations and explore potential effect modification. The absence of multivariate adjustment means the observed associations may be confounded by unmeasured factors such as mental health status, workload, or chronotype.

## 5. Conclusions

Insomnia and excessive daytime sleepiness are highly prevalent among Polish female university students, particularly in the early years of study. Medical students reported lower rates of insomnia but higher levels of sleepiness compared to their peers. Substance use, including alcohol, psychostimulants, and sedatives, showed a significant association with sleep disturbances. Interestingly, the announcement of the COVID-19 pandemic did not influence insomnia rates but was associated with a decrease in daytime sleepiness. These findings underline the need for targeted prevention and intervention strategies to improve sleep health in this population.

## Figures and Tables

**Table 1:** Characteristics of the study group for the total population and by the COVID-19 pandemic.

Variable		Whole Group N (%)	Before Pandemic N (%)	During Pandemic N (%)	Chi^2^	Size Effect	*p*
Age M ± SD		22.03 ± 18.4	22.4 ± 3.0	22.7 ± 4.1	---	–	<0.001
Study year	I	3765 (31.4)	1426 (23.3)	2339 (39.9)	416.69	0.187 #	**<0.001**
	II	2229 (18.6)	1348 (22.0)	881 (15.0)			
	III	2297 (19.2)	1359 (22.2)	938 (16.0)			
	IV	1727 (14.4)	913 (14.9)	814 (13.9)			
	V	1668 (13.9)	924 (15.1)	744 (12.7)			
	VI	302 (2.5)	159 (2.5)	143 (2.5)			
University profile	Medical	2861 (23.9)	1670 (27.3)	1191 (20.3)	78.94	0.082 *	**<0.001**
	Non-medical	9127 (76.1)	4459 (72.7)	4668 (79.7)			
Field of study	Medical	2861 (23.9)	1670 (27.3)	1191 (20.3)	99.34	0.091 #	**<0.001**
	Technical	1607 (13.4)	813 (13.3)	794 (13.6)			
	Humanities	4694 (39.2)	2188 (35.6)	2506 (42.8)			
	Biological	803 (6.7)	420 (6.9)	383 (6.5)			
	Economics	2020 (16.9)	1035 (16.9)	985 (16.8)			
Faculty in the medical field (N = 2861)	Medicine or Dentistry	1486 (51.9)	823 (51.7)	663 (57.7)	30.98	0.105 #	**<0.001**
	Pharmacy	297 (10.3)	216 (13.6)	81 (7.0)			
	Faculty of Health Sciences	960 (33.8)	554 (34.7)	406 (35.3)			
COVID-19 pandemic status announcement	Before pandemic	6129 (51.1)	---	---	---	–	---
	During pandemic	5859 (48.9)	---	---			
Alcohol	Yes	10,479 (87.4)	5568 (90.9)	4911 (83.8)	134.79	0.106 *	**<0.001**
Frequency of use (N = 10,479)	Once a week	2973 (28.4)	1631 (29.4)	1342 (27.3)	20.67	0.045 #	**<0.001**
	Few times a month, less than once a week	3492 (33.3)	1877 (33.8)	1615 (32.7)			
	Not more often than once a month	2830 (27.0)	1485 (26.7)	1345 (27.3)			
	Once in the last 3 months	1184 (11.3)	560 (10.1)	624 (12.7)			
Cannabinoids	Yes	1374 (11.5)	731 (11.9)	643 (11.0)	2.67	0.149 *	0.101
Frequency of use (N = 1374)	Once a week	193 (14.0)	84 (11.2)	109 (17.0)	15.52	0.106 #	**0.001**
	Few times a month, less than once a week	172 (12.5)	387 (52.0)	274 (42.5)			
	Not more often than once a month	362 (26.3)	186 (25.0)	176 (27.4)			
	Once in the last 3 months	661 (48.2)	88 (11.8)	84 (13.1)			
Psychostimulants	Yes	270 (2.3)	130 (2.1)	140 (2.4)	0.98	0.091 *	0.322
Frequency of use (N = 270)	Once a week	39 (14.4)	19 (14.1)	20 (14.6)	0.43	0.039 #	0.934
	Few times a month, less than once a week	39 (14.4)	18 (13.3)	21 (15.3)			
	Not more often than once a month	72 (26.7)	35 (25.9)	37 (27.0)			
	Once in the last 3 months	120 (44.5)	62 (46.7)	58 (43.1)			
Sedatives	Yes	1640 (13.9)	754 (12.5)	886 (15.3)	19.84	0.046 *	**<0.001**
Frequency of use (N = 1640)	Once a week	577 (34.7)	244 (32.6)	333 (37.4)	6.21	0.062 #	0.102
	Few times a month, less than once a week	400 (24.1)	182 (24.2)	218 (24.5)			
	Not more often than once a month	305 (18.4)	142 (19.0)	163 (18.3)			
	Once in the last 3 months	358 (21.6)	181 (24.2)	177 (19.8)			
Using at least 1 of the above substances		10,759 (89.7)	5085 (92.6)	5674 (86.8)	109.01	0.067 *	**<0.001**
The number of used substances (N = 10,759)	0 (10.3)	1229 (10.3)	455 (7.4)	774 (13.2)	119.64	0.102 #	**<0.001**
	1 (67.8)	8123 (67.8)	4327 (70.6)	3796 (64.8)			
	2 (19.1)	2295 (19.1)	1191 (19.4)	1104 (18.8)			
	3 (2.4)	293 (2.4)	139 (2.3)	154 (2.6)			
	4 (0.4)	48 (0.4)	17 (0.3)	31 (0.5)			

Size effect # Cramer’s V, * Phi2.

**Table 2:** Assessment of the effects of university profile, the pandemic, and psychoactive substance use on insomnia using the Athens Insomnia Scale (AIS).

Variable		Whole Group N (%)	Insomnia (%)	No Insomnia N (%)	Chi^2^	Size Effect	*p*
Study year	I	3765 (31.4)	2193 (58.3)	1572 (42.8)	30.97	0.051 #	**<0.001**
	II	2229 (18.6)	1289 (57.8)	940 (42.2)			
	III	2297 (19.2)	1261 (54.9)	1036 (45.1)			
	IV	1727 (14.4)	942 (54.6)	785 (45.5)			
	V	1668 (13.9)	849 (50.9)	819 (49.1)			
	VI	302 (2.5)	167 (55.3)	135 (44.7)			
University profile	Medical	2861 (23.9)	1497 (52.3)	1364 (47.7)	19.46	0.041 *	**<0.001**
	Non-medical	9127 (76.1)	5204 (57.0)	3923 (43.0)			
Field of study	Medical	2861 (23.9)	1497 (52.3)	1364 (47.7)	55.67	0.068 #	**<0.001**
	Technical	1607 (13.4)	930 (57.9)	677 (42.1)			
	Humanities	4694 (39.2)	2749 (58.6)	1945 (41.4)			
	Biological	803 (6.7)	488 (60.7)	315 (39.3)			
	Economics	2020 (16.9)	1037 (51.3)	983 (48.7)			
Faculty in the medical field (N = 2861)	Medicine or Dentistry	1486 (51.9)	749 (50.4)	737 (49.6)	5.54	0.049 #	0.064
	Pharmacy	297 (10.3)	152 (51.2)	145 (48.8)			
	Faculty of Health Sciences	960 (33.8)	530 (55.2)	430 (44.8)			
COVID-19 pandemic status announcement	Before pandemic	6129 (51.1)	3419 (55.8)	2710 (44.2)	0.66	0.003 *	0.798
	During pandemic	5859 (48.9)	3282 (56.0)	2577 (44.0)			
Alcohol	Yes	10,479 (87.4)	5841 (55.7)	4638 (44.3)	0.84	0.008 *	0.361
	No	1509 (13.6)	860 (57.0)	649 (43.0)			
Frequency of use (N = 10,479)	Once a week	2973 (28.4)	1688 (56.8)	1285 (43.2)	2.41	0.015 #	0.491
	Few times a month, less than once a week	3492 (33.3)	1948 (55.8)	1544 (44.2)			
	Not more often than once a month	2830 (27.0)	1572 (55.6)	1258 (44.5)			
	Once in the last 3 months	1184 (11.3)	642 (54.2)	542 (45.8)			
Cannabinoids	Yes	1374 (11.5)	770 (56.0)	604 (44.0)	0.01	0.001 *	0.910
	No	10,614 (88.5)	5931 (55.9)	4683 (44.1)			
Frequency of use (N = 1374)	Once a week	193 (14.0)	101 (52.3)	92 (47.7)	5.51	0.063 #	0.138
	Few times a month, less than once a week	172 (12.5)	106 (61.6)	66 (38.4)			
	Not more often than once a month	362 (26.3)	213 (58.4)	149 (41.6)			
	Once in the last 3 months	661 (48.2)	357 (54.0)	304 (46.0)			
Psychostimulants	Yes	270 (2.3)	169 (62.6)	101 (37.4)	5.03	0.020 *	**0.025**
	No	11,718 (97.7)	6532 (55.7)	5186 (44.3)			
Frequency of use (N = 270)	Once a week	39 (14.4)	24 (61.5)	15 (38.5)	0.72	0.051 #	0.870
	Few times a month, less than once a week	39 (14.4)	23 (59.0)	16 (41.0)			
	Not more often than once a month	72 (26.7)	47 (65.3)	25 (34.7)			
	Once in the last 3 months	120 (44.5)	80 (65.6)	40 (34.4)			
Sedatives	Yes	1640 (13.9)	1318 (80.0)	322 (20.0)	454.82	0.194 *	**<0.001**
	No	10,327 (86.1)	5372 (52.0)	4955 (48.0)			
Frequency of use (N = 1640)	Once a week	577 (34.7)	502 (87.0)	75 (13.0)	31.07	0.138 #	**<0.001**
	Few times a month, less than once a week	400 (24.1)	226 (74.1)	79 (25.9)			
	Not more often than once a month	305 (18.4)	316 (79.0)	84 (21.0)			
	Once in the last 3 months	358 (21.6)	267 (74.6)	91 (25.4)			
Using at least 1 of the above substances		10,759 (89.7)	6057 (56.3)	4702 (43.7)	6.79	0.041 *	**0.009**
The number of used substances	0	1229 (10.3)	644 (52.4)	585 (47.6)	186.59	0.112 #	**<0.001**
	1	8123 (67.8)	4277 (52.7)	3846 (47.3)			
	2	2295 (19.1)	1544 (67.3)	751 (32.7)			
	3	293 (2.4)	200 (68.3)	93 (31.7)			
	4	48 (0.4)	36 (75.0)	12 (25.0)			

Size effect # Cramer’s V, * Phi2.

**Table 3:** Assessment of the effects of university profile, the pandemic, and psychoactive substance use on daytime sleepiness using the Epworth Sleep Scale (ESS).

Variable		Whole Group N (%)	Excessive Sleepiness (%)	No Sleepiness N (%)	Chi^2^	Size Effect	*p*
Study year	I	3765 (31.4)	1079 (28.7)	2686 (71.3)	12.89	0.033 #	**0.024**
	II	2229 (18.6)	633 (28.4)	1596 (71.6)			
	III	2297 (19.2)	643 (28.0)	1654 (72.0)			
	IV	1727 (14.4)	445 (25.8)	1282 (74.2)			
	V	1668 (13.9)	412 (24.7)	1256 (75.3)			
	VI	302 (2.5)	82 (27.2)	220 (72.8)			
University profile	Medical	2861 (23.9)	901 (31.5)	1960 (68.5)	30.11	0.051 *	**<0.001**
	Non-medical	9127 (76.1)	2393 (26.2)	6734 (73.8)			
Field of study	Medical	2861 (23.9)	901 (31.5)	1960 (68.5)	36.44	0.055 #	**<0.001**
	Technical	1607 (13.4)	447 (27.8)	1160 (72.2)			
	Humanities	4694 (39.2)	1213 (25.8)	3481 (74.2)			
	Biological	803 (6.7)	229 (28.5)	574 (71.5)			
	Economics	2020 (16.9)	504 (25.0)	1516 (75.0)			
Faculty in the medical field (N = 2861)	Medicine or Dentistry	1486 (51.9)	453 (30.5)	1033 (69.5)	1.77	0.026 #	0.411
	Pharmacy	297 (10.3)	98 (33.0)	199 (67.0)			
	Faculty of Health Sciences	960 (33.8)	315 (32.8)	645 (67.2)			
COVID-19 pandemic status announcement	Before pandemic	6129 (51.1)	1837 (30.0)	4292 (70.0)	39.16	0.057 *	**<0.001**
	During pandemic	5859 (48.9)	1457 (24.9)	4402 (75.1)			
Alcohol	Yes	10,479 (87.4)	2915 (27.8)	7564 (72.2)	4.83	0.021 *	**0.028**
	No	1509 (13.6)	379 (25.1)	1130 (74.9)			
Frequency of use (N = 10,479)	Once a week	2973 (28.4)	879 (29.6)	2094 (70.4)	11.22	0.033 #	**0.011**
	Few times a month, less than once a week	3492 (33.3)	938 (26.9)	2554 (73.2)			
	Not more often than once a month	2830 (27.0)	801 (28.3)	2029 (71.7)			
	Once in the last 3 months	1184 (11.3)	296 (24.9)	888 (75.1)			
Cannabinoids	Yes	1374 (11.5)	434 (31.6)	940 (68.4)	13.15	0.034 *	**<0.001**
	No	10,614 (88.5)	2860 (26.9)	7754 (73.1)			
Frequency of use (N = 1374)	Once a week	189 (14.0)	49 (26.4)	140 (73.6)	2.98	0.046 #	0.393
	Few times a month, less than once a week	168 (12.5)	49 (29.7)	119 (70.3)			
	Not more often than once a month	358 (26.3)	115 (32.3)	243 (67.7)			
	Once in the last 3 months	659 (48.2)	214 (32.5)	445 (67.5)			
Psychostimulants	Yes	270 (2.3)	97 (35.9)	173 (64.1)	9.89	0.029 *	**0.002**
	No	11,718 (97.7)	3197 (27.3)	8521 (72.7)			
Frequency of use (N = 270)	Once a week	39 (14.4)	13 (33.3)	26 (66.7)	0.28	0.028 #	0.979
	Few times a month, less than once a week	39 (14.4)	14 (35.9)	25 (64.1)			
	Not more often than once a month	72 (26.7)	27 (37.5)	45 (62.5)			
	Once in the last 3 months	120 (44.5)	42 (35.3)	78 (64.7)			
Sedatives	Yes	1640 (13.9)	513 (31.6)	1127 (68.5)	16.02	0.037 *	**<0.001**
	No	10,327 (86.1)	2770 (26.8)	7557 (73.2)			
Frequency of use (N = 1640)	Once a week	577 (34.7)	184 (31.9)	393 (68.1)	2.77	0.041 #	0.428
	Few times a month, less than once a week	400 (24.1)	136 (34.0)	264 (66.0)			
	Not more often than once a month	305 (18.4)	88 (28.9)	217 (71.2)			
	Once in the last 3 months	358 (21.6)	106 (29.6)	252 (70.4)			
Using at least 1 of the above substances		10,759 (89.7)	2988 (27.8)	7771 (72.2)	4.58	0.032 *	**0.032**
The number of used substances	0	1229 (10.3)	306 (24.9)	923 (75.1)	38.67	0.061 #	**<0.001**
	1	8123 (67.8)	2151 (26.5)	2151 (26.5)			
	2	2295 (19.1)	714 (31.1)	1581 (68.9)			
	3	293 (2.4)	101 (34.5)	192 (65.5)			
	4	48 (0.4)	22 (45.8)	26 (52.2)			

Size effect # Cramer’s V, * Phi2.

## Data Availability

The data presented in this study are available on request from the corresponding author.
